# The impact of thymectomy in subgroups of Myasthenia gravis patients: a single center longitudinal observation

**DOI:** 10.1186/s42466-023-00252-w

**Published:** 2023-06-15

**Authors:** Hruy Menghesha, Michael Schroeter, Christopher Nelke, Tobias Ruck, Georg Schlachtenberger, Clara Welskop, Amina Camo, Matthias Heldwein, Gerardus Bennink, Thorsten Wahlers, Servet Bölükbas, Fabian Doerr, Khosro Hekmat

**Affiliations:** 1 Department of Thoracic Surgery, University Medical Center Essen - Ruhrlandclinic, Tüschener Weg 40, 45239 Essen, Germany; 2 Faculty of Medicine, Department of Cardiothoracic Surgery, University of Cologne, University Hospital Cologne, Kerpener Strasse 62, 50937 Cologne, Germany; 3 Faculty of Medicine, Department of Neurology, University of Cologne, University Hospital Cologne, Kerpener Strasse 62, 50937 Cologne, Germany; 4 Faculty of Medicine, University of Cologne, Joseph-Stelzmann-Strasse 20, 50931 Cologne, Germany; 5 Department of Neurology, Medical Faculty, Heinrich Heine University Düsseldorf, Dusseldorf, Germany

**Keywords:** Myasthenia gravis, Multimodal therapy, Thymectomy, Antibodies

## Abstract

**Background:**

Myasthenia gravis (MG) is a rare neuromuscular disorder. Symptoms can range from ptosis only to life threatening myasthenic crisis. Thymectomy is recommended for anti-acetylcholine receptor-antibody positive patients with early-onset MG. Here, we investigated prognostic factors shaping therapeutic outcomes of thymectomy to improve patient stratification.

**Methods:**

We retrospectively collected single-center data from a specialized center for MG from all consecutive adult patients that underwent thymectomy from 01/2012 to 12/2020. We selected patients with thymoma-associated and non-thymomatous MG for further investigations. We analyzed the patient collective regarding perioperative parameters in relation to the surgical approach. Furthermore, we investigated the dynamics of the anti-acetylcholine receptor-antibody titers and concurrent immunosuppressive therapies, as well as the therapeutic outcomes in dependence of clinical classifications.

**Results:**

Of 137 patients 94 were included for further analysis. We used a minimally invasive approach in 73 patients, whereas 21 patients underwent sternotomy. A total of 45 patients were classified as early-onset MG (EOMG), 28 as late-onset MG (LOMG) and 21 as thymoma-associated MG (TAMG). The groups differed in terms of age at diagnosis (EOMG: 31.1 ± 12.2 years; LOMG: 59.8 ± 13.7 years; TAMG: 58.6 ± 16.7 years; p < 0.001). Patients with EOMG and TAMG were more often female than patients in the LOMG group (EOMG: 75.6%; LOMG: 42.9%; TAMG: 61.9%; p = 0.018). There were no significant differences in outcome scores (quantitative MG; MG activities of daily living; MG Quality of Live) with a median follow-up of 46 months. However, Complete Stable Remission was achieved significantly more frequently in the EOMG group than in the other two groups (p = 0.031). At the same time, symptoms seem to improve similarly in all three groups (p = 0.25).

**Conclusion:**

Our study confirms the benefit of thymectomy in the therapy of MG. Both, the concentration of acetylcholine receptor antibodies and the necessary dosage of cortisone therapy show a continuous regression after thymectomy in the overall cohort. Beyond EOMG, groups of LOMG and thymomatous MG responded to thymectomy as well, but therapy success was less pronounced and delayed compared to the EOMG subgroup. Thymectomy is a mainstay of MG therapy to be considered in all subgroups of MG patients investigated.

## Background

Myasthenia gravis (MG) is a rare neuromuscular disorder with a prevalence of 77 to 167 per million people living in Europe [1] and an incidence between 4 and 18 cases per million person-years [2] characterized by muscle fatigue manifesting as diplopia, ptosis, bulbar and/or limb weakness [3, 4]. MG can be clinically stratified into various subgroups, which in turn result in different therapeutic strategies [4]. Depending on the age of the patient at initial diagnosis, antibody-status, clinical severity of symptoms, and comorbidities, conservative treatment options range from symptomatic therapy with acetylcholine esterase inhibitors to immunosuppressive therapy with cortisone and other immunosuppressants to administration of immunoglobulins and plasma exchange [5]. Until recently, only the presence of a thymic tumor was a class I indication for resection of thymic tissue in patients with MG, because randomized controlled data were lacking, although thymectomy appeared to be a proven remedy in various cases [6, 7]. It was not until the 2016 randomized controlled trial published in the New England Journal of Medicine that has proven the efficacy of thymectomy in patients aged 18–60 years with MGFA stage II-IV, non-thymomatous, anti-AchR-ab positive MG [8]. Although the first randomized controlled data were not available until 2016, thymectomy has been conducted for immunomodulatory indications for more than a century. Since Ferdinand Sauerbruch performed the first thymectomy in a patient with hyperthyroidism and myasthenia gravis in 1912 [9], more diverse surgical options have been developed. Retrospective data analyses suggested that patients before the age of 18 years and beyond the age of 65 years, that are seronegative or show isolated ocular symptoms (MGFA I), could also benefit from thymectomy with low perioperative risk [10–13]. Although the detection of a thymoma requires an oncologic R0 resection, the thymic tissue should also be resected radically for non-thymomatous myasthenia gravis to improve symptoms effectively [14]. Incomplete resection of thymic tissue resulted in persistence of symptoms and the need for reoperation in various studies [15–17]. Even though the general doctrinal opinion was the necessity of transsternal radical thymectomy, median sternotomy is a highly invasive and disfiguring procedure. The introduction of various minimally invasive approaches improved the feasibility of thymecytomy with comparable radicality [18]. Here, we report retrospective data of the past 10 years from our Myasthenia gravis center aiming to provide further data on factors that might prove advantageous or disadvantageous for therapeutic outcomes after thymectomy.

## Methods

We collected retrospective fully anonymized data from our institutional thymectomy database. For further analysis, we divided patients into groups according to surgical approach (minimally invasive versus total sternotomy) and according to the clinicopathologic subgroups [thymoma-associated MG (TAMG) versus early-onset MG (EOMG) versus late-onset MG (LOMG)], which were examined with respect to perioperative endpoints and functional disease-specific endpoints. The age of 50 was chosen as the age limit defining the EOMG and LOMG groups [19].

### Inclusion and exclusion criteria

As one of 13 integrated Myasthenia gravis centers in Germany, the therapy of patients with MG is carried out in close cooperation with the Department of Neurology and depending on the etiology as a paraneoplastic syndrome, with the Department of Oncology. All 137 consecutive patients that underwent thymectomy from 01/2012 to 12/2021 at our institution were screened for our study. Resected tissue was analyzed concerning presence of malignant cells, resection margin (R0, R1 or R2), and presence of lymphofollicular hyperplasia and stage of involution. Patients that did not present with MG were excluded from further analysis. Thus, of the initial 137 patients, 94 patients remained with whom further analyses were conducted.

### Clinical imaging

All patients diagnosed with MG and presented to us by the diagnosing neurologist for surgical therapy underwent thoracic computed tomography (CT) using contrast agent, which was intended to detect a possible tumorous structure in the thymus on the one hand and to relate the individual anatomic structures on the other. In rare cases, when the patient was referred to us by a resident colleague with a radiological incidental finding, imaging was performed by thoracic magnetic resonance imaging (MRI).

### Blood tests

**Fig. 1:**
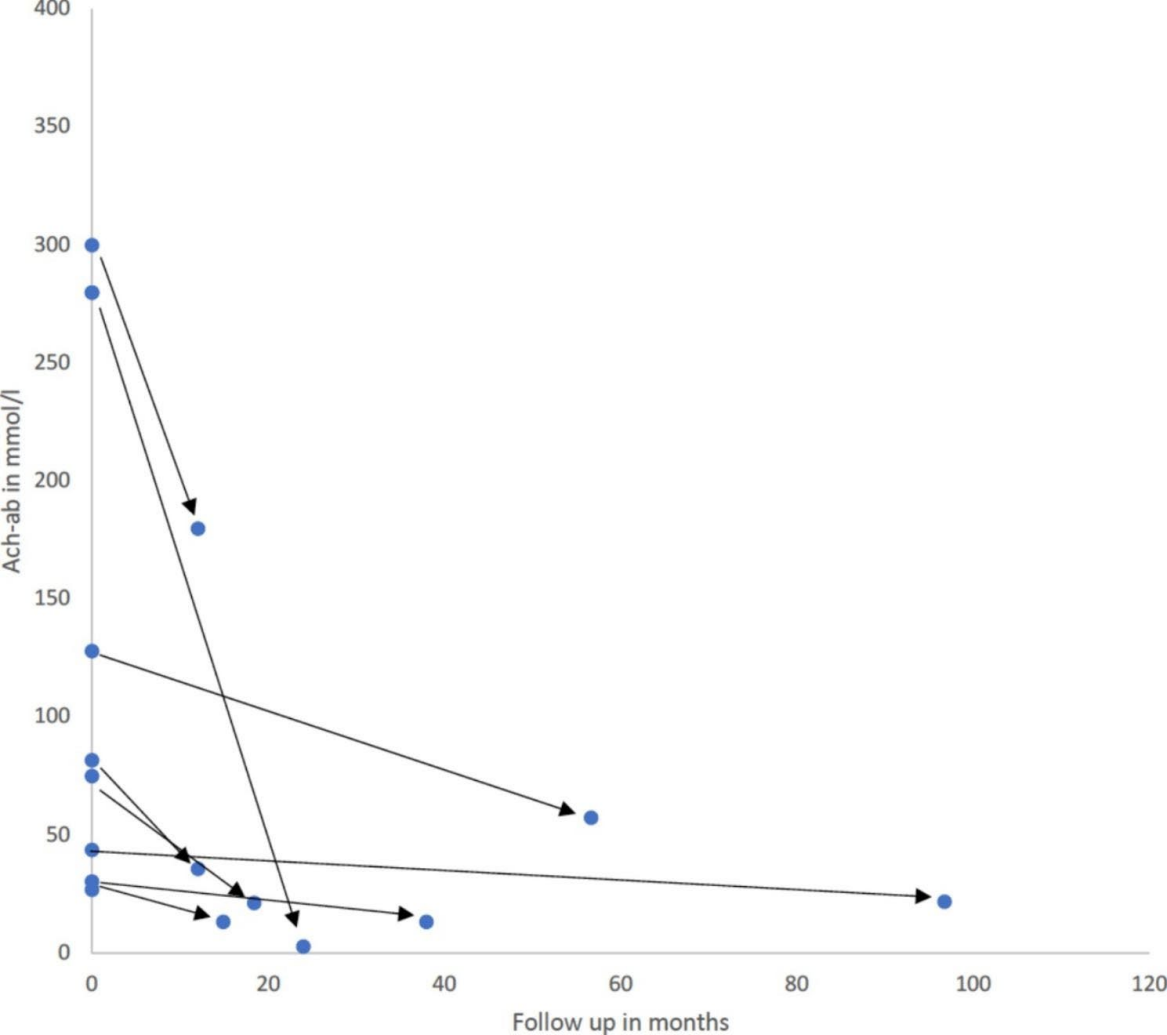
Follow up reduction of AchR-ab concentration after thymectomy

Most patients underwent blood testing for disease-specific autoantibodies. These included the acetylcholine-receptor antibodies (AchR-ab), muscle-specific kinase antibodies (MuSK-ab), and the titin antibodies (T-ab). During the performed follow-up, the appropriate antibodies were determined in all presented patients. The evolution of the concentration of AchR-ab over time after thymectomy was transferred into a scatter plot and the trend was shown by means of graphs (see Fig. 1).

### Neurological examinations

All patients were examined and diagnosed preoperatively by a neurologist specialized in MG. Comparable and reproducible examinations included in our investigation were the Simpson test, the MG Quality of life test (MG-QoL), and the MG Activities of daily living test (MG-ADL). During regular follow-up, we additionally evaluated the quantitative MG Score (qMG), the Postintervention Status Score (PIS), and the MG Composite Score (MG-Comp.).

### Histopathological examination

The histopathological examination of the resected thymic tissue was conducted by local pathologist. The analysis included the description of the detected thymomas according to Masaoka-Koga and WHO stage in terms of the predominant cell population.

If no malignancy was found, the pathologists distinguished between the detection of lymphofollicular hyperplasia, thymitis and age-appropriate atrophy. In some cases, immunohistochemical analyses were performed.

### Surgical procedure

The included patients were all operated on by a senior surgeon at our department. The surgical approach was either a videoassisted-thoracoscopic surgical approach, or a full sternotomy. The decision which approach was chosen depended on the patient’s physical constitution, comorbidities, and the size of the expected thymic mass.

### Statistical analysis

Continuous variables, if normally distributed, were summarized as means ± standard deviations (SDs) and analyzed by independent-samples T-test or paired Student’s t-test; if not, were summarized as median [interquartile range (IQR)] and analyzed by Mann–Whitney test or Wilcoxon signed-rank test. The doses of cortisone use during the course, as well as the course of AchR antibodies after thymectomy, were each plotted in a scatter plot and a vector of development was implemented into the coordinate system. Baseline-characteristics between the clinicopathologic subgroups were first analyzed with one-way ANOVA depending on normal distribution of the interval-scaled parameters. If significant differences appeared, variables were analyzed using post hoc analysis. A p-value < 0.05 was accepted as statistically significant.

## Results

A total of 94 of initially 137 patients were eligible for further analysis. For subgroup analysis the study population was divided into two groups according to the surgical approach [minimal-invasive approach (MIC, n = 73) versus full sternotomy (FS, n = 21)]. Subsequently, a subgroup analysis regarding clinicopathological staging was performed. The clinical categories EOMG (n = 45), LOMG (n = 28) and TAMG (n = 21) were distinguished.

### Baseline characteristics of the whole study population

Baseline characteristics for the whole cohort are presented in Table 1. Mean age of patients was 45.8 ± 19.63 years. The proportion of female patients was 62.8%. Preoperatively, 83.9% of all patients were taking pyridostigmine, 72.2% cortisone, and 28.7% another immunosuppressant. Most patients were preoperatively in MGFA stage II (57.3%) and 78.7% of all patients diagnosed with myasthenia gravis showed acetylcholine receptor antibodies. Titin antibodies were detected in 19.8% of all patients and only 3.8% of patients showed muscle-specific kinase antibodies (MuSK-Ab). Among patients with thymoma-associated MG, WHO stage B1 was the most common histological stage, accounting for 38.1%. None of those patients showed recurrence during follow up.

**Table 1:** Baseline characteristics of all 94 patients

	Total (n = 94)
**Demographic data**	
Age (years)	45.8 ± 19.6
Female (%)	62.8
BMI (kg/m^2^)	27.4 ± 6.7
**Surgical Approach (%)**	
MIC	77.7
Sternotomy	22.3
**Preoperative medication (%)**	
Pyridostigmin	83.9
Cortisone	72.2
Other Immunosuppressants	28.7
Myocphenolatmophetil	3.8
Azathioprin	27.5
Rituximab	2.2
**Preoperative IVIg-therapy (%)**	6.1
**Clinical classification (%)**	
TAMG	22.3
EOMG	47.9
LOMG	29.8
**Preoperative MGFA-Stage (%)**	
I	16
IIA	36.2
IIB	21.1
IIIA	7.4
IIIB	5.3
IVA	5.3
IVB	3.2
V	1.1
**Perioperative antibody positive (%)**	
Acetylcholine	78.7
MuSK	3.8
Titin	19.8
ICU-stay (days)	1 [0;16]
**Hospital stay (days)**	8.5 ± 8.4
**Thymoma**	
**Masaoka-Koga Stage (%)**	
I	18.0
IIA	2.0
III	1.0
**WHO Stage (%)**	
Type A	4.8
Type AB	28.6
Type B1	38.1
Type B2	19.0
Type B3	9.5
**Follow up in months (WHO-Stage)**	**months**
Type A	7.8 ± 0.0
Type AB	45.2 ± 44.2
Type B1	24.9 ± 20.3
Type B2	23.4 ± 19.4
Type B3	46.4 ± 21.9

### Subgroup analysis surgical approach

A total of 21 patients underwent surgery via median sternotomy, while 73 of the 94 patients underwent minimally invasive surgery. The differences of the mean age, as well as the distribution with regard to the gender of the patients, and the BMI showed no statistical significance. Patients operated on via MIC access were operated on for 106.7 ± 34.9 min, while those whose sternum was fully opened were operated on for 94.95 ± 29.98 min. This difference was not found to be statistically relevant (p = 0.166). However, patients who underwent sternotomy had to be hospitalized a mean time of 3 days longer (MIC: 6 [1;37]; Sternotomy: 9 [6;65]; p = 0.002). The patients showed no statistically significant differences in respect to perioperative complications including the need for reintubation and pneumonia development. In addition, patients in both groups showed improvement in functional limitation and concomitant quality of life as measured by the MG-ADL and the MG-QoL with no statistically significant difference (Table 2).

### Subgroup analysis of clinicopathological staging

The TAMG group was comparable to the LOMG group in respect to age, while patients in the EOMG were younger on average (EOMG: 31.1 ± 12.2 years; LOMG: 59.8 ± 13.7 years; TAMG: 58.6 ± 16.7 years; p-value (EOMG vs. TAMG) < 0.001; p-value (LOMG vs. TAMG) = 1.0).

In accordance with the literature, the analysis of gender distribution revealed that female patients were significantly more frequent in the EOMG group than in the LOMG group (p = 0.014; M_Diff_ = 0.33, 95%-CI [0.05, 0.6]). However, the difference to the TAMG group did not show statistical significance. Also, patients in the TAMG group were more often female than in the LOMG group, however, without reaching statistical significance.

The preoperative severity of neurological symptoms measured by MGFA stage showed no differences among the three groups. Although we observed a trend for TAMG patients to be more severely affected than patients of the other two groups, results failed statistical significance (p = 0.051). In the preoperative blood examination, the frequency of detection of AchR-ab showed no difference among the three groups. However, Titin-ab were detected more frequently in the TAMG group than in the other two groups (p < 0.001). Bonferroni-corrected post-hoc analysis revealed a significant difference of preoperative presence of Titin antibodies in EOMG-group compared to LOMG-group (p = 0.032; M_Diff_ = 0.252, 95%-CI [0.02,0.49]) and TAMG-group (p < 0.001; M_Diff_ = 5.02, 95%-CI [0.24,0.77]), but not between LOMG-group and TAMG-group (p = 0.105; M_Diff_ = 0.249, 95%-CI [-0.04,0.53]. The mean time to follow-up was 44 months in the EOMG group, 45 months in the LOMG group, and 49 months in the TAMG group (p = 0.699). The MG-ADL, MG-QoL, qMG, and MG composite showed no statistically significant differences between the three groups at follow-up (MG-ADL: p = 0.298; MG-QoL: p = 0.242; qMG: p = 0.776; MG-Composite: p = 0.407). However, analysis of the PIS showed that patients in the EOMG group were more likely to demonstrate Complete Stable Remission (CSR). Overall, 18.8% of patients with an EOMG reached a CSR, whereas only 4.8% of patients in the LOMG and none of the patients in the TAMG group had a CSR. At the same time, 84.6% of patients in the TAMG group showed MM3 stage, while the proportion was only 50% in the EOMG group. Bonferroni-corrected post-hoc analysis revealed a significant difference between PIS in the EOMG-group and the TAMG-group (p = 0.049; M_Diff_ = 1.31, 95%-CI [0.0039,2.6]) (Table 3). A total of 27.7% of the cohort were already receiving nonsteroidal immunosuppression such as azathioprine, mycophenolate mophetil, or rituximab preoperatively (Table 4). The distribution within the subgroups showed no significant difference (p = 0.98).

**Table 2:** Subgroup analysis distributed by surgical approach

	Total (n = 94)	MIC (n = 73)	Sternotomy (n = 21)	p-value
Demographic data				
Age (years)	45.8 ± 19.6	45.0 ± 18.3	48.6 ± 23.9	0.15
Female (%)	62.8	60.3	71.4	0.45
BMI (kg/m^2^)	27.4 ± 6.7	27.3 ± 7.1	27.7 ± 5.5	0.782
**Operating time (min.)**				
	104.1 ± 34.1	106.7 ± 34.9	94.9 ± 29.9	0.166
**ICU stay (days)**				
	1 [0;16]	1 [0;4]	1 [0;16]	0.746
**Inhospital stay (days)**				
	6 [1;65]	6 [1;37]	9 [6;65]	**0.002**
**Perioperative complications**				
PMC (%)	4.3	4.1	4.8	1.0
Reintubation (%)	2.1	2.7	0	1.0
Pneumonia (%)	5.3	4.1	9.5	0.31
**Outcome Analysis**				
MGQoL preop.	21.5 [0;36]	21.5 [0;36]	14.5 [0;29]	0.49
MGQoL postop.	3.5 [0;28]	6 [0;28]	0.5 [0;28]	0.76
MG-ADL preop.	5 [0;15]	5 [0;11]	5 [0;15]	0.213
MG-ADL postop.	2 [0;16]	2 [0;12]	0 [0;16]	0.833

**Table 3:** Preoperative characteristics distributed by clinical-pathogenetic aspects

	Total (n = 94)	EOMG (n = 45)	LOMG (n = 28)	TAMG (n = 21)	p-value
Age (years)					
	45.8 ± 19.6	31.1 ± 12.2	59.8 ± 13.7	58.6 ± 16.7	**< 0.001**
Female (%)					
	62.8	75.6	42.9	61.9	**0.018**
BMI (kg/m^2^)					
	27.4 ± 6.7	25.0 ± 4.9	30.3 ± 9.0	28.4 ± 4.9	**0.003**
Surgical appr. (%)					**0.002**
Sternotomy	22.3	20	7.1	47.6	
MIC	77.7	80	92.9	52.4	
Preop. MGFA (%)					0.051
I	16	18.2	14.3	15.8	
IIA	36.2	47.7	32.1	21.1	
IIB	21.1	18.2	25.0	26.3	
IIIA	7.4	11.4	10.7	0	
IIIB	5.3	2.3	7.1	10.5	
IVA	5.3	0	7.1	15.1	
IVB	3.2	2.3	0	10.5	
V	1.1	0	3.6	0	
Preop. Antib. (%)					
AchR	78.7	68.9	92.9	81	0.05
Titin	21.8	2.8	28.0	52.9	**< 0.001**
MuSK	3.8	2.7	3.6	6.3	0.83
Preop. Med. (%)					
Pyridostigmin	84.0	88.9	85.7	71.4	0.19
Cortison	72.3	68.9	85.7	61.9	0.14
Other	27.7	22.2	42.9	19	0.98

**Age:**

Bonferroni-corrected post-hoc analysis revealed a significant difference between age of the EOMG-group and the LOMG-group (p < 0.001; M_Diff_ = 28.7, 95%-CI [20.6,36.8]) and the EOMG-group and the TAMG-group (p < 0.001; M_Diff_ = 27.1, 95%-CI [18.7,36.4]) but not between the LOMG-group and the TAMG-group (p = 1.0; M_Diff_ = 1.2, 95%-CI [-8.5,10.9])

**Female gender:**

Bonferroni-corrected post-hoc analysis revealed a significant difference between presence of female gender in the EOMG-group and the LOMG-group (p = 0.014; M_Diff_ = 0.33, 95%-CI [0.05,0.6])

**BMI:**

Bonferroni-corrected post-hoc analysis revealed a significant difference between BMI in the EOMG-group and the LOMG-group (p = 0.003; M_Diff_ = 5.33, 95%-CI [1.5,8.9])

**Titin:**

Bonferroni-corrected post-hoc analysis revealed a significant difference of preoperative presence of Titin antibodies in EOMG-group compared to LOMG-group (p = 0.032; M_Diff_ = 0.252, 95%-CI [0.02,0.49]) and TAMG-group (p < 0.001; M_Diff_ = 5.02, 95%-CI [0.24,0.77]), but not between LOMG-group and TAMG-group (p = 0.105; M_Diff_ = 0.249, 95%-CI [-0.04,0.53]

**Table 4:** Outcome analysis distributed by clinical-pathogenetic aspects

	Total(n = 94)	EOMG(n = 45)	LOMG(n = 28)	TAMG(n = 21)	p-value
Follow up (m)					
	46 [12;151]	44 [12;151]	45 [14;74]	49 [18;107]	0.699
**MG-ADL postOP**					
	2 [0;16]	3 [0;12]	0 [0;8]	2 [0;16]	0.298
**MG-QoL**					
	3,5 [0;28]	5 [0;27]	2 [0;17]	7 [0;28]	0.242
**qMG**					
	1 [0;18]	1 [0;17]	0 [0;15]	2 [0;18]	0.776
**MG-Composite**					
	1 [0;27]	1.5 [0;21]	0 [0;8]	2 [0;27]	0.407
**AchR (nmol/l)**					
	4,5 [0;674]	3 [0;674]	4,5 [0,12;85]	5.4 [1,8;273]	0.42
**PIS**					**0.031**
** CSR**	10.6	18.8	4.8	0	
** MM0**	9.1	15.6	4.8	0	
** MM1**	12.1	6.3	19.0	15.4	
** MM2**	4.5	9.4	0	0	
** MM3**	63.6	50	71.4	84.6	
					0.25
** Improved**	56.9	51.6	61.9	61.5	
** Unchanged**	29.2	38.7	19	23.1	
** Worse**	13.8	9.7	19	15.4	

PIS

Bonferroni-corrected post-hoc analysis revealed a significant difference between PIS in the EOMG-group and the TAMG-group (p = 0.049; M_Diff_ = 1.31, 95%-CI [0.0039,2.6])

### Immunotherapeutic effect of thymectomy

**Fig. 2:**
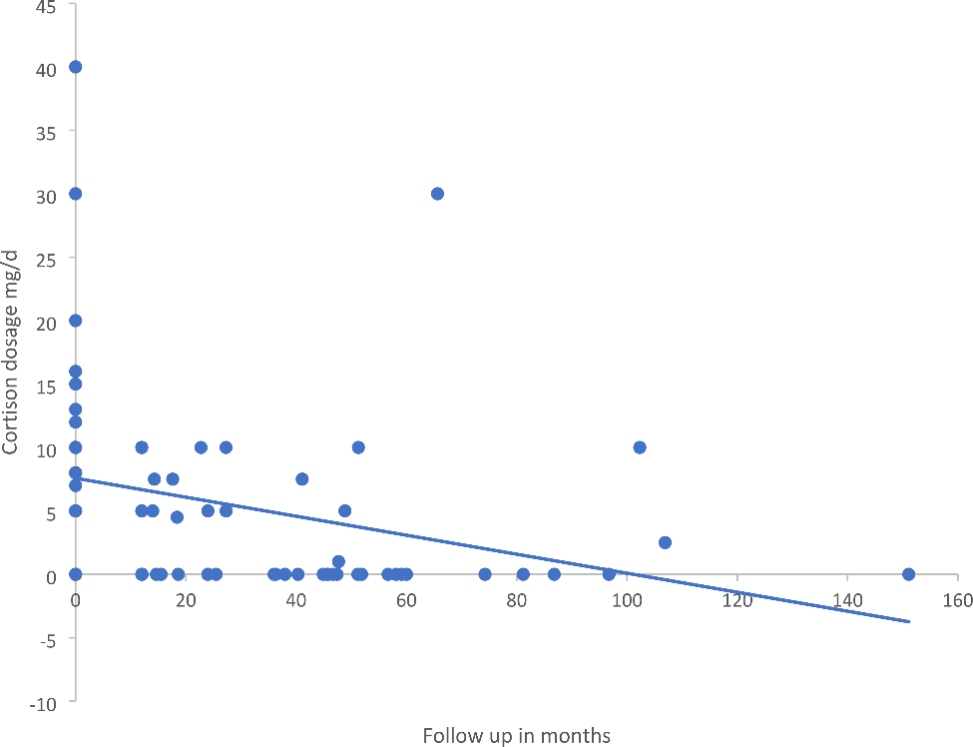
Presentation of the reduction of the necessary cortisone intake

Several pharmacological studies, as well as the MGTX trial have proposed the therapeutic effect on the cortisone dose as additional outcome parameter[8]. Regarding the whole cohort, cortisone dosage decreased over time between time point 0 (t0; before thymectomy) and time point of follow up (t1) (r = 5 [-20; 62.5] mg/d) see Fig. 2. To analyze the individual dynamic of cortisone, we calculated according to the equation ((Cortisone dosage t0 [mg prednisolone equivalent]) – (cortisone dosage t1 [mg prednisolone equivalent])) / time to follow up [months]. Here we observed for the corresponding subgroups: overall cohort: 7.2 mg/d / 45.7 months = 0.16 mg/d/month; EOMG: 5 mg/d / 43.9 months = 0.11 mg/d/month; LOMG: 8 mg/d / 44.9 months = 0.17 mg/d/month; TAMG: 0.5 mg/d / 48.9 months = 0.01 mg/d/month.

Hence, cortisone sparing was successful in both in EOMG and LOMG subcohorts to a similar extent but was almost never observed in the TAMG group (Fig. 2).

Although absolute AchR-ab titers vary considerably on an interindividual level the individual decline of titers can be associated with clinical improvement. We compared AchR-ab titers in a subgroup of patients with titers available before thymectomy and at follow up (Fig. 1). Unequivocally, AchR-ab titer dynamics declined over time. At first glance, the higher the titer the steeper was the fall of titers observed after thymectomy (Fig. 1).

## Discussion

MG is a rare disease of the neuromuscular junction. Although our pathophysiological understanding of the disease has made substantial progress, clinical data are scarce about the translation of therapeutic concepts into real world patient management. The care of MG in Germany is largely organized in specialized centers as initiated by the German Myasthenia Gravis Foundation. Thymectomy plays a crucial part in the multimodal therapy of MG. In EOMG and LOMG, indication of thymectomy is not an oncological but an immunotherapeutic one that demands a strict understanding of the pathophysiological concept, first class evidence for its prospective benefit among the individual subgroup and a constant survey of positive effects on the disease course, and complications of surgery.

### The cohort

Inherent to retrospective cohorts, there is a risk of substantial selection bias by referral (here: referral to thymectomy). However, demographic data of our cohort suggest a representative profile as compared to national cohorts [20, 21]. The population falls apart in young EOMG and older LOMG and TAMG patients with relative sparing of the 5th decade. Female gender predominated the EOMG group whereas in LOMG and TAMG, gender distribution is balanced. Positive Titin-ab are indicative of LOMG and TAMG, but do not discriminate between those groups.

### Is minimally invasive surgery appropriate for immunomodulatory indications?

As evidenced from the results, the sternotomy and MIC groups show no significant difference in terms of perioperative parameters, with comparable baseline characteristics. Comparing sternotomy and a minimally invasive approach, the latter was associated with a shorter in-hospital stay. This advantage might not only lead to an increase in patient satisfaction, but also to a reduction in costs. At the same time, the clinical outcome and thus the benefit of the intervention were comparable.

The debate regarding the optimal surgical approach persists, not only due to the fact that the thymus spans two anatomic regions, cervical and mediastinal [22, 23]. Transsternal thymectomy is a form of surgical resection of thymic tissue initiated in the first half of the 20th century for the treatment of non-thymomatous MG as well as for the treatment of thymic tumors [14]. Although the only randomized-controlled data to date demonstrated the effectiveness of thymectomy using only a transsternal approach [8], and this surgical method was declared by Alfred Jaretzki [24] to be necessary for the sufficient therapy of MG, retrospective data show the improvement of symptoms and a reduced rate of generalized disease when the procedure is performed early with minimally invasive surgical methods [10, 25]. Minimally invasive thymectomy combines the transcervical approach, video-assisted/robotic-assisted thoracoscopic approach, and subxiphoid approach. Videoscopy can support minimally invasive cervical, subxiphoid, and thoracoscopic approaches and facilitate resection of thymic tissue and mediastinal adipose tissue [26, 27].

Interestingly, none of the 21 TAMG patients showed recurrence after initial resection, which does not correspond to the frequency of approximately 8% reported in the literature [28]. At the same time, the evaluation should not ignore the fact that the follow-up of the patients shows a marked inhomogeneity with regard to the time interval.

### Does age at onset or etiology predict better outcome?

The average follow-up in the three groups EOMG, LOMG and TAMG was comparable regarding the length of time after thymectomy. Considering the trajectory of qMG, postoperative MG-ADL, MG composite, and MG-QoL score, there were no significant differences among the three groups. However, our study demonstrates that the patients with an EOMG achieved CSR significantly more often than the patients in the other two groups, which is congruent with the results of the investigation of Na et al. [29].

Although thymectomy is currently recommended only for EOMG patients up to 50 years of age, the results of the MGTX trial show positive effects up to 65 years of age, i.e. also in LOMG patients [8, 30]. Typically, patients with LOMG present histopathologically with thymic involution, whereas patients in EOMG more commonly present with follicular hyperplasia. However, patients in either group may present with the pathologic feature of the other group, demonstrating the difficulty of establishing clear cut-offs [11]. Although the latency to improve symptoms after thymectomy is prolonged in elderly patients, retrospective data show a benefit to patients after both sternotomy and minimally invasive thymectomy [31].

### Immune effects of thymectomy: cortisone sparing effects

As known from several pharmacological studies as well as the MGTX trial, the cortisone sparing effects are a commonly accepted readout to quantify immunotherapeutic benefits in MG [32]. In our cohort, cortisone dosage overall declined over time. On an individual basis, the LOMG group appears to present a similar cortisone-sparing effect as in the EOMG group. Still, immunological effects may be systematically underestimated in our cohort: First, thymectomy is performed after stabilization of MG before operation. Consequently, cortisone doses are likely already decreased prior to the operation introducing a potential bias when comparing cortisone usage across groups. Moreover, in contrast to the MGTX study, we commonly use azathioprine to taper down prednisolone before thymectomy, if necessary. As such, a fourth of all patients received azathioprine at the time of thymectomy. The cortisone tapering effect is already well known [33]. With such strategies already in place at the time of thymectomy, the (additional) absolute cortisone sparing after thymectomy may be underappreciated as reported in other settings, approximating a “ceiling effect” in this particular readout.

### Immune effects of thymectomy: decline of AchR-ab titers

The patient collective showed regredient concentrations of measured AchR-ab with no significant difference in measured absolute concentration between the three groups EOMG, LOMG, and TAMG (Fig. 2; Table 3). On an individual level, however, vectors of AchR-ab dynamics show unequivocally a decline of titers after thymectomy (Fig. 1).

In cross sectional studies there is a weak correlation of absolute AchR-ab titers and clinical severity of MG [34–36]. In accordance, we found a wide range of ab levels in our cohort. In longitudinal studies, however, though not undebated, the dynamics of AchR-ab titer on an individual level better correlate with clinical remission [37–39]. In a small subgroup of our cohort the dynamics of AchR-ab titers are available that show an unequivocal fall of titers after thymectomy; enough to hypothesize that the success of thymectomy may be assessed by falling AchR-ab titers in further clinical studies. At the same time, a retrospective analysis from China published in 2021 showed that there was no direct correlation between the concentration of AchR-ab and clinical symptoms in a similarly small cohort of 67 patients [27]. This was in agreement with the results of a case series from 1978, but it must be taken into account that the study time points were very inhomogeneous and in the range of weeks after surgical resection of the thymus [28]. Succinctly, there remains uncertainty regarding the value of AchR-ab as biomarker underlying the need for further studies in this context.

As can be seen in Table 4, 3.8% of MusK ab positive patients are distributed in the three groups EOMG, LOMG and TAMG in our investigation. These patients showed simultaneous detection of either AchR ab or titin ab. As we know from previous investigation, the pure MusK ab positive patients do not seem to benefit from thymectomy [19, 40]. The group of pure MusK ab positive patients is incredibly interesting and should also be the subject of multicentric studies with regard to the benefit of thymectomy.

### Immune effects of thymectomy: proportion of patients achieving CSR

Based on the follow-up described, the EOMG group appears to be significantly more likely to achieve CSR after thymectomy than the TAMG group, which is consistent with the existing literature [8, 41]. TAMG patients tended to be more severely affected and in that clinical subgroup, therapeutic success was somewhat limited. Consequently, TAMG demands consequent and long-lasting immunosuppression including immunosuppressive and monoclonal antibody therapies [42, 43] apart from a sufficient oncological therapy.

### Limitations

This study was limited by its retrospective nature; due to the limited number of patients included in this study, it is difficult to draw conclusions about the overall efficacy of the intervention. Furthermore, only 8 patients had consistent AchR-ab concentrations preoperatively and at follow up time, which may limit the statistical power of the study.

Follow-up time, especially radiological follow-up in the TAMG group, was inconsistent. Of course, the interpretation of the cortisone-sparing effect of thymectomy against the background of preoperative use of nonsteroidal immunosuppressants is also limited. Finally, the sample size of the study was small, which may limit the generalizability of the results.

## Conclusion

Awareness of the possibility of immunologic intervention by removal of the thymus gland in patients with EOMG is now widespread. In addition to the class I recommendations for EOMG and the oncological indication in TAMG, it remains to be investigated whether thymectomy appears less successful for LOMG patients. This study suggests a comparable benefit from thymectomy in terms of symptom control, and cortisone sparing effects, for EOMG and LOMG patients. Eventually, thymectomy increases the chance of achieving complete clinical remission without ongoing immunosuppressive medication. We hypothesize that these effects are more generalized in MG patients, beyond the EOMG subgroup.

## Data Availability

The data underlying this article will be shared on reasonable request to the corresponding author.

## Abbreviations

AchR-ab: Acetylcholine receptor antibodies
CI: Confidence interval
CT: Computed tomography
EOMG: Early Onset Myasthenia Gravis
FS: Full sternotomy
IQR: Interquartile range
LOMG: Late Onset Myasthenia Gravis
MG: Myasthenia gravis
MG-ADL: Myasthenia Gravis activities of daily living Score
MGFA: Myasthenia Gravis Foundation of America
MG-QoL: Myasthenia Gravis Quality of Live Score
MIC: Minimally invasive surgery
MRI: Magnetic resonance imaging
MuSK-ab: Muscle-specific kinase antibodies
PIS: Postintervention status
qMG: Quantitative Myasthenia Gravis score
SD: Standard deviation
T-ab: Titin antibodies
TAMG: Thymoma-associated
